# P-45. A Phase 3, Randomized Trial Investigating the Safety, Tolerability, and Immunogenicity of V116, an Investigational Adult-Specific Pneumococcal Conjugate Vaccine, in Pneumococcal Vaccine-Naïve Adults 18–64 Years of Age with Increased Risk for Pneumococcal Disease

**DOI:** 10.1093/ofid/ofae631.252

**Published:** 2025-01-29

**Authors:** Paul Scott, Jayani Pathirana, Akira Kato, Richard Tytus, Carlos M Perez, Nigel Leslie Gilchrist, Hidemi Kanou, Kwang Ha Yoo, Grzegorz Kania, Michael Nissen, Michael Livingston, Amy Falk Russell, Doreen Fernsler, Muhammad Waleed, Jianing Li, Ulrike K Buchwald, Heather L Platt

**Affiliations:** Merck & Co., Inc., Rahway, NJ, USA, Rahway, New Jersey; Merck & Co., Inc., Rahway, NJ, USA, Rahway, New Jersey; Shimonoseki Medical Center, Shimonoseki, Japan, Shimonoseki, Yamaguchi, Japan; McMaster University, Hamilton, Ontario, Canada; San Sebastian University, Santiago, Chile, Santiago, Region Metropolitana, Chile; CGM Research Trust, Christchurch, New Zealand, Christchurch, Canterbury, New Zealand; Kurume University School of Medicine, Fukuoka, Japan, Fukuoka, Fukuoka, Japan; Konkuk University Medical Center, Seoul, Republic of Korea, Seoul, Seoul-t'ukpyolsi, Republic of Korea; Medical University of Lublin, Lublin, Poland, Lublin, Lubelskie, Poland; The Prince Charles Hospital, Chermside, Australia, Chermside, Queensland, Australia; SKY Integrative Medical Center, Ridgeland, Mississippi, USA, Ridgeland, Mississippi; Merck & Co., Inc., Rahway, NJ, USA, Rahway, New Jersey; Merck & Co., Inc., Rahway, NJ, USA, Rahway, New Jersey; Merck & Co., Inc., Rahway, NJ, USA, Rahway, New Jersey; Merck & Co., Inc., Rahway, NJ, USA, Rahway, New Jersey; Merck & Co., Inc., Rahway, NJ, USA, Rahway, New Jersey; Merck & Co., Inc., Rahway, NJ, USA, Rahway, New Jersey

## Abstract

**Background:**

Adults with certain underlying chronic medical conditions are at increased risk of pneumococcal disease (PD). V116 is an investigational, 21-valent, adult-specific pneumococcal conjugate vaccine (PCV) containing the most prevalent serotypes (STs) associated with PD in adults from regions with established pediatric vaccination programs. The Phase 3 STRIDE-8 study (NCT05696080) evaluated the safety and tolerability of V116 in adults 18–64 years of age at increased risk of PD. Immunogenicity of V116 was compared with sequential administration of 15-valent PCV (PCV15) followed by 23-valent pneumococcal polysaccharide vaccine (PPSV23).

**Figure:**
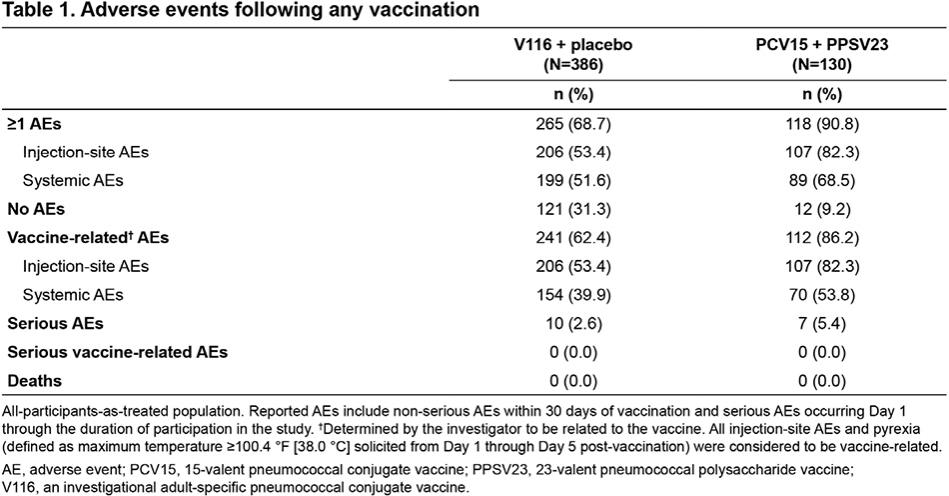

**Methods:**

Pneumococcal vaccine-naïve participants with ≥1 underlying chronic medical conditions (including diabetes mellitus, heart disease, kidney disease, liver disease, and lung disease) at increased risk of PD were randomized 3:1 to receive one dose of V116 on Day 1 followed by placebo at Week 8 or one dose of PCV15 on Day 1 followed by one dose of PPSV23 at Week 8. Safety was evaluated as the proportion of participants with adverse events (AEs). Immunogenicity was assessed by serotype-specific opsonophagocytic activity (OPA) geometric mean titers (GMTs) and immunoglobulin G (IgG) geometric mean concentrations (GMCs) for STs in V116 at baseline (Day 1) and 30 days post-vaccination (Day 30 for V116 + placebo and Week 12 for PCV15 + PPSV23).

**Figure:**
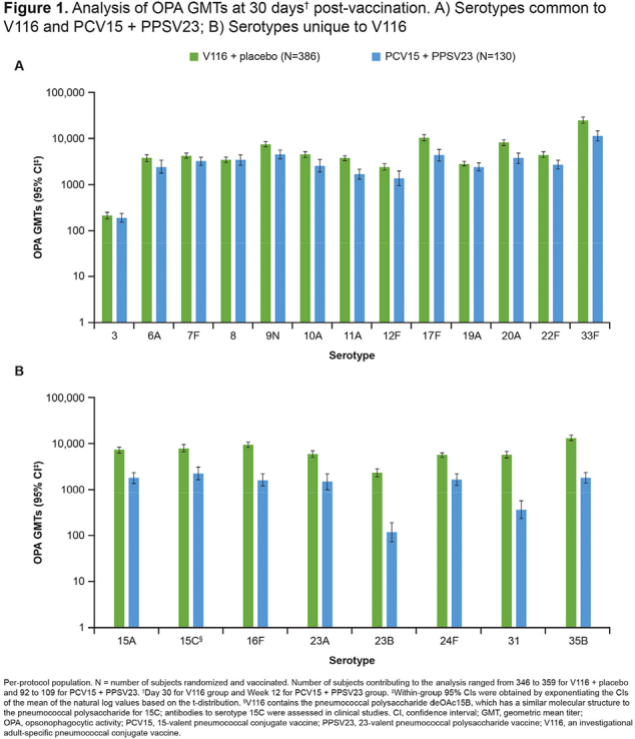

**Results:**

Of 518 participants randomized, 516 were vaccinated and received either V116 (n=386) or PCV15 (n=130) on Day 1; 96.7% of participants completed the trial. One or more AEs occurred in 265 (68.7%) and 118 (90.8%) participants vaccinated with V116 + placebo or PCV15 + PPSV23, respectively (**Table 1**). V116 was immunogenic for all 21 STs based on OPA GMTs, with comparable responses to PCV15 + PPSV23 for the 13 STs common to V116 and PCV15 + PPSV23, and higher responses for the eight STs unique to V116 (**Figure 1**). IgG GMCs were consistent with OPA GMTs (**Figure 2**).

**Figure:**
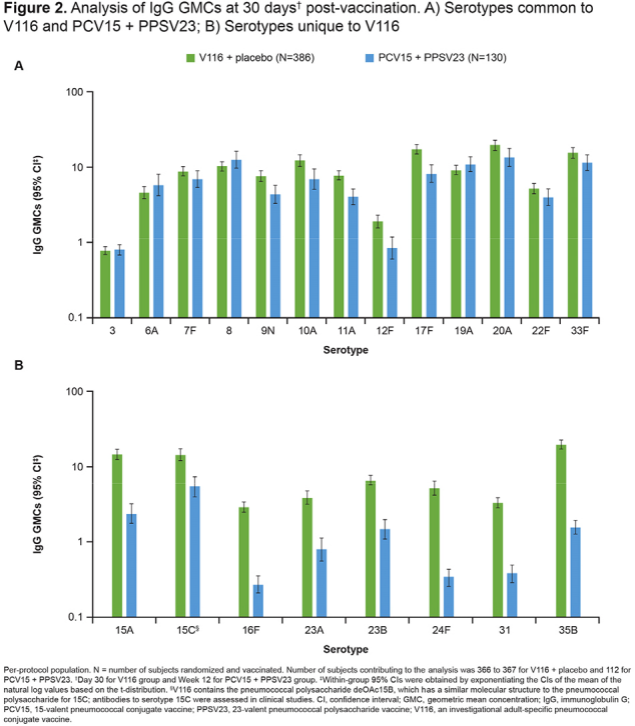

**Conclusion:**

V116 is well tolerated and immunogenic in adults 18–64 years of age at increased risk of PD, with comparable immune responses to PCV15 + PPSV23 for common STs and higher immune responses for unique STs. These findings support V116 as a novel population-specific vaccine for the prevention of PD in adults with chronic medical conditions at increased risk of PD.

**Disclosures:**

**Paul Scott, MD**, Merck Sharp & Dohme LLC, a subsidiary of Merck & Co., Inc., Rahway, NJ, USA: Employee|Merck Sharp & Dohme LLC, a subsidiary of Merck & Co., Inc., Rahway, NJ, USA: Stocks/Bonds (Private Company) **Jayani Pathirana, MBBS**, Merck Sharp & Dohme LLC, a subsidiary of Merck & Co., Inc., Rahway, NJ, USA: Employee|Merck Sharp & Dohme LLC, a subsidiary of Merck & Co., Inc., Rahway, NJ, USA: Stocks/Bonds (Private Company) **Michael Nissen, MD**, CSL Seqirus: Education|GSK: Education|Pfizer: Education **Amy Falk Russell, MS**, Merck Sharp & Dohme LLC, a subsidiary of Merck & Co., Inc., Rahway, NJ, USA: Employee|Merck Sharp & Dohme LLC, a subsidiary of Merck & Co., Inc., Rahway, NJ, USA: Stocks/Bonds (Private Company) **Doreen Fernsler, BS**, Merck Sharp & Dohme LLC, a subsidiary of Merck & Co., Inc., Rahway, NJ, USA: Employee|Merck Sharp & Dohme LLC, a subsidiary of Merck & Co., Inc., Rahway, NJ, USA: Stocks/Bonds (Private Company) **Muhammad Waleed, PhD**, Merck Sharp & Dohme LLC, a subsidiary of Merck & Co., Inc., Rahway, NJ, USA: Employee|Merck Sharp & Dohme LLC, a subsidiary of Merck & Co., Inc., Rahway, NJ, USA: Stocks/Bonds (Private Company) **Jianing Li, PhD**, Merck Sharp & Dohme LLC, a subsidiary of Merck & Co., Inc., Rahway, NJ, USA: Employee|Merck Sharp & Dohme LLC, a subsidiary of Merck & Co., Inc., Rahway, NJ, USA: Stocks/Bonds (Private Company) **Ulrike K. Buchwald, MD**, Merck Sharp & Dohme LLC, a subsidiary of Merck & Co., Inc., Rahway, NJ, USA: Employee|Merck Sharp & Dohme LLC, a subsidiary of Merck & Co., Inc., Rahway, NJ, USA: Stocks/Bonds (Private Company) **Heather L. Platt, MD**, Merck Sharp & Dohme LLC, a subsidiary of Merck & Co., Inc., Rahway, NJ, USA: Employee|Merck Sharp & Dohme LLC, a subsidiary of Merck & Co., Inc., Rahway, NJ, USA: Stocks/Bonds (Private Company)

